# Agricultural work and reduced circulating uric acid are both associated with initial hospital admission for Parkinson’s disease

**DOI:** 10.1007/s00702-019-02119-4

**Published:** 2019-12-14

**Authors:** Hanxiang Liu, Xianwen Wei, Wen Yang, Gavin P. Reynolds

**Affiliations:** 1 Department of Neurology, Puer People’s Hospital, Puer, Yunnan China; 2 Biomolecular Sciences Research Centre, Sheffield Hallam University, Howard Street, Sheffield, S1 1WB UK

**Keywords:** Parkinson’s disease, Uric acid, Pesticide exposure, Farming, Oxidative damage, Neurodegeneration

## Abstract

Monoamine oxidase type B inhibitors act in Parkinson’s disease (PD) via potentiation of dopamine, but may also have neuroprotective effects by reducing oxidative damage. Oxidative damage is also a feature of environmental toxins, including pesticides, that are an established risk factor for PD. Another risk factor is low circulating uric acid (UA), which may relate to UA being the major endogenous antioxidant in the human body. We have undertaken a study of 192 initial admissions for PD in a general hospital neurology department in a partly rural region of Southern China to determine if there is an increased rate of PD in agricultural workers who have a high risk of exposure to pesticides, and how it may relate to deficits in UA. We found a disproportionately high number of agricultural workers admitted with PD (66.7% vs. 54.3% of all neurology admissions) and that PD subjects have a substantial reduction in UA. This is further reduced in agricultural workers and thus may contribute to the increased vulnerability of this group to PD.

## Introduction

The introduction of deprenyl (selegiline) was an important development in the treatment of Parkinson’s disease (PD), pioneered in the UK by the clinical group of Gerald Stern working with the biochemical and pharmacological expertise of Merton Sandler’s research team (Lees et al. 1977). Deprenyl is a selective inhibitor of monoamine oxidase (MAO) type B which avoids the toxic effects of non-selective MAO inhibitors when combined with levodopa (Elsworth et al. 1978), the long-established treatment for PD. Deprenyl potentiates the effect of levodopa-derived dopamine by inhibiting its breakdown; however, it may also have protective effects in reducing oxidative free radicals that can contribute to neurodegenerative processes (Clow et al. 1991). These observations have contributed to understanding the role of oxidative damage in the pathogenesis of PD, indicating the importance of endogenous and exogenous antioxidants and free radical scavengers in neuroprotection (Gaki and Papavassiliou 2014; Wei et al. 2018).

The major circulating antioxidant in the human body is uric acid (UA) (Bowman et al. 2010). Although elevated UA increases the risk of cardiovascular disease and stroke, it appears to have neuroprotective properties as it is found to be reduced in patients with several neurodegenerative disorders, including Alzheimer’s (Khan et al. 2016; Mullan et al. 2018) and Parkinson’s diseases (Shen et al. 2013), as well as being associated with outcome of ischaemic stroke (Liu et al. 2018).

A further risk factor associated with PD, albeit with limited direct causal evidence (Breckenridge et al. 2016), is exposure to environmental toxins including pesticides (Yan et al. 2018). One such compound, rotenone, is used to model PD in rodents; its mechanism of action involves the production of toxic reactive oxygen species (ROS) (Xiong et al. 2012). While not necessarily the only mechanism involved in the toxic effect of pesticides, this process is likely to be a component of the pathogenic mechanism of PD associated with many such environmental toxins (Heusinkveld et al. 2014).

One group of individuals at high risk of exposure to pesticides is that of agricultural workers; neurotoxic effects of such exposure are relatively frequent in farmers in Southern China (Hu et al. 2015). This risk may be particularly great among the rural farming community of China’s Yunnan province, which is relatively poorly educated and economically disadvantaged. We aimed to investigate whether there is evidence that this group has an increased risk of PD and/or an earlier disease onset. We also aimed to determine the association of circulating UA with PD in agricultural and non-agricultural workers, using a sample of patients with vascular dementia (VD) for comparison, in whom we previously found no significant abnormality in UA (Liu et al. 2020).

## Methods

The study cohort was drawn from subjects admitted to the Pu’er People’s Hospital, Department of Neurology, for the initial investigation associated with symptoms of PD. All patients included underwent neuroimaging, the great majority magnetic resonance imaging (MRI), to exclude cerebral vascular disease and other diseases resulting in a Parkinson syndrome, following which diagnosis of PD (ICD10: G20.x00) was made on the basis of symptoms which include slowness of movement (bradykinesia), resting tremor, rigidity, postural instability and clinical history, according to the Movement Disorder Society Clinical Diagnostic Criteria (Li et al. 2017). From this neurological examination, a Hoehn and Yahr (HY) stage score of I, II, or III was obtained. Further exclusion criteria were a history of epilepsy, kidney disease, liver disease or multiple organ failure, cancer, gout, substance abuse, prior severe mental illness, or psychoactive drug treatment. This yielded a PD series of 192 subjects. Current cigarette use was recorded. All subjects had undergone routine blood testing prior to initiation of antiparkinsonian drug treatment; fasting plasma samples were collected before 8 am by venipuncture in heparin–lithium anticoagulant tubes. After centrifugation, plasma samples were aliquoted in polypropylene tubes for biochemical analyses using routine assays from which UA data were collected.

Data from a previously studied sample (Liu et al. 2020) of 124 patients meeting diagnostic criteria for VD, including mixed dementia, were used for comparison of UA results and their relationship with agricultural work. Details of recent admissions to the hospital neurology department were used for comparison with the proportion of agricultural workers in the study.

Statistical analysis was performed using SPSS v21. Results for UA were tested for normal distribution (Kolmogorov–Smirnoff test); as they deviated significantly from normality all statistical testing was undertaken with log-transformed UA data. *T* tests, univariate analysis of variance, Chi-square test, and Pearson correlation were used to compare samples and determine correlations. The study was approved by the Hospital Research Ethics Committee; as an anonymized retrospective study, requirement for informed consent was waived.

## Results

Of the PD subjects, exactly twice as many were agricultural workers as not (Table 1). This contrasted with the ratio in neurological admissions; in a period of 10 months (January–October 2019), there were 9012 admissions to the Neurology Department of Pu’er People’s Hospital, of whom 4892 (54.3%) were agricultural workers. PD shows a significantly different distribution (Chi-square = 11.63, *p* < 0.001) with an odds ratio of 1.68 (CI 1.24–2.28).

**Table 1:** Details of subjects studied including plasma uric acid concentrations

	Parkinson’s disease			Vascular dementia		
Agricultural work	Yes	No	*p*	yes	no	*p*
Sex M/F	56/72	35/29	0.152	34/16	58/16	0.211
Age (years)	62.28 ± 9.30	66.80 ± 9.56	**0.002**	67.00 ± 9.69	70.82 ± 10.47	**0.042**
Age of onset (years)	59.08 ± 9.76	62.72 ± 9.96	**0.017**			
HY stage I	39	17	0.078			
HY stage II	69	28				
HY stage III	16	16				
Cigarette smoking (Y/N)	9/108	10/50	0.068	16/34	30/44	0.334
Uric acid (µmol/L)	273 ± 83 (125)	313 ± 77 (62)	0.005*	334 ± 115 (46)	380 ± 109 (66)	0.021*

Bold values are statistically significant

Data are mean ± standard deviation, or number. *p* values from *t* tests or Chi-square tests, except *ANOVA controlling for age and sex, with UA data log-transformed

Comparing the PD subjects, the agricultural workers were younger, reflecting an earlier age of onset (Table 1). This age difference was also apparent in the VD sample. No significant difference in severity between the PD groups was apparent from the overall distribution of the HY stage scores, although there was a lower proportion of stage III subjects in the agricultural workers (Chi-square = 5.08, *p* = 0.024). A higher proportion of females were present in the agricultural group, although this was not statistically significant. There were a smaller proportion of current smokers in the agricultural workers with PD which did not reach statistical significance.

UA was found to be reduced in the PD group (286 ± 83 µmol/L) in comparison with both the VD sample (361 ± 113 µmol/L; *F* = 19.92, *p* < 0.001) and a previously studied (Liu et al. 2020) and approximately age-matched (62.5 ± 10.3 years; *n* = 79) healthy control sample (348 ± 86 µmol/L; *F* = 31.12, *p* < 0.001), controlling for age and sex. UA concentrations were lower in agricultural workers in both PD and VD samples (Table 1). A univariate analysis of the PD and VD data together indicated that diagnosis (PD or VD), work type (agricultural or not), and sex each had significant associations with UA (each *p* = 0.001 or less), with no significant interactions, nor an influence of age as a covariate. This analysis remained qualitatively unchanged when analyzing only non-smoking subjects. Univariate analysis of the PD data alone showed that sex and work type remained strongly significant influences on UA, with no significant effect of HY stage.

UA correlated positively with age of onset in the PD group, controlling for sex (*r* = 0.165, *p* = 0.024). In male and female groups separately, this effect did not reach significance. The significant correlation was lost in the agricultural workers group (*r* = 0.050, *p* = 0.58), but was greater in the non-rural group (*r* = 0.276, *p* = 0.032). No such correlation between age and UA was found in the VD sample (*r* = − 0.017, *p* = 0.86).

## Discussion

We found that PD subjects admitted to the neurology department of a general hospital had a far higher proportion of agricultural workers than a sample of all neurological admissions. This finding is consistent with the hypothesis that agricultural work, perhaps due to exposure to environmental toxins such as pesticides, is a strong risk factor for the development of PD. While the number of PD subjects who smoked was relatively few, the lack of an appropriate comparison group and an adequate sample size prevented an assessment of whether our data were consistent with evidence that non-smokers have an increased risk of PD (Breckenridge et al. 2016; Li et al. 2015).

While there was an earlier age of PD onset in agricultural workers, the fact that those with VD were also younger than the non-rural VD patients suggested a systematic bias, possibly reflecting differences in age distribution resulting from different mortality rates. There was no overall difference in disease severity determined by the HY scale between the agricultural and non-agricultural PD patients. However, the smaller proportion of HY scale III subjects in the generally younger former group does not support a risk of earlier, or more aggressive, PD development in agricultural workers.

Lower concentrations of UA were also associated with PD, in comparison with both a healthy control sample and the VD patient group. This adds to the substantial evidence that low UA is a risk factor for PD. Lower UA was also, and independently, associated with agricultural work. It is unclear why there appears to be, from the results of both PD and VD samples, a consistent effect in agricultural workers of lower UA. Circulating UA is determined by both genetics and diet, although the genetic influence reportedly contributes more to the variance than individual dietary factors (Major et al. 2018). Nevertheless, dietary differences between the groups may well be important in influencing UA levels (Schmidt et al. 2013). One hypothetical mechanism is that, in a rural population, a relatively reduced intake of animal protein could result in lower circulating UA. It is unlikely to be an effect of pesticide exposure, which has been reported to increase blood concentrations of UA (Lee et al. 2013).

We were unable to find evidence in support of the recent finding in a smaller sample (Zhong et al. 2018) of a relationship between HY stage and UA levels. This may reflect other differences between the studies; our sample was generally less severe, with no patients of HY stage greater than 3, and untreated. Another study of 80 PD patients also showed greater reduction in UA with disease severity and, in contrast to Zhong et al. (2018), with levodopa treatment (Vieru et al. 2016).

That UA has a protective effect is further indicated by the positive correlation with age of onset, supporting recent findings (Oh et al. 2019) that higher concentrations may delay the degenerative process and hence the emergence of symptoms. That this was only identified in the subjects who were not agricultural workers is notable; it may reflect the effect of a UA threshold which is not reached in most agricultural workers with further reduced UA concentrations, or it may indicate that other factors, such as pesticide exposure, are more important in determining risk of PD in this group.

This study is limited by its cross-sectional and retrospective nature as a survey of PD hospital admissions, rather than being population-based and prospective, indicating that identified associations cannot be concluded to be risk factors. Importantly, the current study does not allow us to determine whether the additional UA deficit in agricultural workers is additional to a risk associated with, for example, pesticide use, or whether it is the main factor responsible for the PD risk in this group. A healthy, or non-neurological, control group differentiated into agricultural and non-agricultural workers would have been a valuable comparator. Furthermore, there may still be unidentified artifactual influences responsible for the higher admission rate of agricultural workers with PD. While the sample size is substantial in comparison with several other studies, the power to investigate relationships between subgroups is limited. A larger sample would, for example, have permitted an assessment of whether the protective effect of cigarette smoking influences the associations of PD with agricultural work or low UA. Whether serum UA is a useful indicator of antioxidant activity in the brain is unclear, although blood and CSF UA are reported to be correlated (Bowman et al. 2010). Direct determination of pesticide exposure would be essential in drawing any conclusions relating to their role as causative factors for PD. Furthermore, determining differences between agricultural and non-agricultural groups in diet and other measures associated with antioxidant activity, as well as determining inflammatory markers and genetic risk factors including family history, would also be valuable in future studies.

In conclusion, we have identified a substantially increased rate of hospital admissions for PD from agricultural workers, consistent with an increase in disease risk in this subgroup, possibly driven by exposure to pesticides. We also confirm in these early stage, untreated PD patients, a reduction in serum UA, another risk factor for the disease, and that there is an additional reduction in UA associated with agricultural work. Thus, this greater reduction in UA may be an additional risk factor associated with the development of PD in agricultural workers. Further controlled studies are needed to determine if this is sufficient to explain the excess of PD in agricultural workers, or whether it is additional to other factors such as pesticide exposure.
